# Current definition of acute kidney injury actually identifies a heterogenous group of patients with elevated serum creatinine and reduced urine output

**DOI:** 10.1186/s13054-020-02968-6

**Published:** 2020-05-24

**Authors:** Yanxiao Chen

**Affiliations:** Department of Evidence-Based Medicine, Affiliated Dongyang Hospital of Wenzhou Medical University, Dongyang, Zhejiang People’s Republic of China

Dear Editor,

I read with great interest in the study by Hoste and colleagues [1], which tried to identify novel biomarkers for early diagnosis of acute kidney injury (AKI). However, I must point out that seeking novel biomarkers for the early identification of AKI by comparing the diagnostic performance of these novel biomarkers (or in combination) against AKI definition based on serum creatinine and urine output is invalid. The definition of AKI based on serum creatinine and urine output is for the ease of clinical use, but it is not a gold standard for AKI definition. Actually, the AKI is not a disease with a solid pathological signature that can be definitely confirmed as that for tumors [2]. AKI based on the current definition encompasses a heterogeneous population. For example, the kidney will stop to produce urine due to circulatory insufficiency; this should be better defined as the success of the kidney rather than failure because the kidney tries to restore effective circulatory volume by reducing urine output. Another situation is that the kidney is intrinsically injured by uncontrolled inflammatory response and relevant oxidative stress. In this case, the reduction in urine output and increased serum creatinine is caused by true kidney injury [3]. In Hoste’s study, the biomarkers such as urinary cell cycle arrest biomarkers and chitinase 3-like protein 1 (CHI3L1) are well-established renal injury biomarkers, but they appeared to perform poorly in the study. The primary reason for this is because the working criteria for AKI (i.e., the Kidney Disease: Improving Global Outcomes (KDIGO)) are not suitable for the identification of novel renal biomarkers. AKI identified by KDIGO actually comprises a heterogeneous group of patients with increased serum creatinine and reduced urine output, and thus, subphenotypes should be identified for the purposes of clinical practice and study designs [4]. With respect to the combination of biomarkers to improve the diagnostic performance of novel biomarkers, I suggest that biomarkers can be combined by building a multivariable regression model with the presence/absence of AKI as the response variable and all biomarkers to be combined as predictors [5]. After model fitting, a weight will be assigned to each biomarker, which will give a risk score for each biomarker. By summing up all points associated with all biomarkers, a total score can be obtained, and a higher score will indicate a higher risk of AKI.

## Data Availability

No data for the work.
